# Supplementary material for: Effect of eucalyptus wood-based compost application rates on soil chemical properties in semi-organic avocado plantations, Limpopo province, South Africa
Source: PLoS One. 2023 Feb 7;18(2):e0265728. doi: 10.1371/journal.pone.0265728 (PMC9904473; doi:10.1371/journal.pone.0265728)
Supplement: Attachment — Submitted filename: Avo Response to reviewers.doc [file pone.0265728.s001.doc]

Response to reviewers

| **Reviewer’s comments** | **Author’s response** |
| --- | --- |
| Please ensure that your manuscript meets PLOS ONE's style requirements, including those for file naming. | The PLOS ONE’s style was adopted in preparing the manuscript |
| In your Methods section, please provide additional information regarding the permits you obtained for the work. Please ensure you have included the full name of the authority that approved the field site access and, if no permits were required, a brief statement explaining why. | The additional information was added as was suggested |
| We note that the grant information you provided in the ‘Funding Information’ and ‘Financial Disclosure’ sections do not match. When you resubmit, please ensure that you provide the correct grant numbers for the awards you received for your study in the ‘Funding Information’ section. | No specific grant was given for this study |
| Thank you for stating the following in the Funding Section of your manuscript:  “This research was funded by National Research Fund (NRF) and ZZ2 Bertie van Zyl (Pty) Ltd, South Africa.”  We note that you have provided funding information that is not currently declared in your Funding Statement. However, funding information should not appear in the Acknowledgments section or other areas of your manuscript. We will only publish funding information present in the Funding Statement section of the online submission form.  Please remove any funding-related text from the manuscript and let us know how you would like to update your Funding Statement. Currently, your Funding Statement reads as follows:  “he funders had no role in study design, data collection and analysis, decision to publish, or preparation of the manuscript”  Please include your amended statements within your cover letter; we will change the online submission form on your behalf. | The research did not receive any specific grant although the first author was supported by the NRF and the ZZ2 Bertie van Zyl (Pty) Ltd, South Africa but not under any specific grant as was stated |
| Please include your full ethics statement in the ‘Methods’ section of your manuscript file. In your statement, please include the full name of the IRB or ethics committee who approved or waived your study, as well as whether or not you obtained informed written or verbal consent. If consent was waived for your study, please include this information in your statement as well. | The ethics statement was written in the Methods section as was indicated |
| Please include a copy of Table 7. | Table 7 was inserted as suggested |
| **Rviewer #1** |  |
| Line 114-115: Is there any field capacity data? If there is, can you add it? | The field capacity information was included as suggested. See the yellow colour in the attached manuscript |
| Line 196: under t ha ? which application? | The missing tonnage was added- see the correction in yellow |
| Line 310: Additions of articles titled as "The First Effect of Eucalyptus camaldulensis Leaves and Eucalyptol Additions on Soil Carbon Mineralization" and "Short-Term Eucalyptus and Phragmites Biochar’s Efficiency in Mineralization of Soil Carbon" can help to explain how eucalyptus compost affect on soil organic carbon accumulation in soil. | The additional literature was added as was suggested. See yellow marks in the attached manuscript. The given sources were consulted. |
| Due to the high salinity of compost, addition of this compost into soil may have directly affect the activity of soil microorganisms in this study. Soil biological properties are directly linked with soil chemical properties. Is there any suggestion for reducing the salinity of this compost while it was produced? In addition to that, is there any data about the EC of irrigation water in this study? Is it possible that salinity of irrigation water may have increased soil salinity? All application rates of this compost significantly increased soil chemical properties compared to control. However, EC can be a big problem in plant production. Based on EC data in this study, I think 5 t/ha application rate of eucalyptus compost is more optimum dose for improving soil chemical properties rather than higher doses. | Yes, we agree with the reviewer’s suggestion but the EC of the soils were < 1 dS/m (the maximum salinity level where salinity problem may occur in plant production) in 0. 5 and 10 t/ha eucalyptus-wood-based compost (See table 5 and 9). Hence application rates up to 15 t/ha may not be such problematic salinity according to our findings.  The EC of the irrigation water was added as was suggested. |

Regards,

Prof. A Manyevere
